# Neural basis of impaired narrative discourse comprehension in prodromal and mild dementia with lewy bodies

**DOI:** 10.3389/fnagi.2022.939973

**Published:** 2022-09-15

**Authors:** Anaïs Falque, Mélanie Jordanis, Lionel Landré, Paulo Loureiro de Sousa, Mary Mondino, Emmanuelle Furcieri, Frédéric Blanc

**Affiliations:** ^1^ICube Laboratory and FMTS (Fédération de Médecine Translationnelle de Strasbourg), Team IMIS, Université de Strasbourg, Strasbourg, France; ^2^Geriatrics Department, CM2R (Memory Resource and Research Centre), University Hospitals of Strasbourg, Strasbourg, France

**Keywords:** prodromal dementia with lewy bodies, narrative discourse, executive functions, voxel-based morphometry, striatum

## Abstract

Narrative discourse (ND) comprehension is a complex task that implies not only linguistic abilities but also other cognitive abilities, including efficient executive functioning. An executive dysfunction has been described in dementia with Lewy bodies (DLB) from the early stage. Here, we question the link between executive dysfunction in DLB and narrative comprehension. The aim of our study was to evaluate ND comprehension and to investigate the neuroanatomical basis for its impairment in the early stage of DLB. DLB patients (*N* = 26) and controls (*N* = 19) underwent the ND comprehension test of the Montreal Protocol for Evaluation of Communication (MEC). An additional, qualitative analysis was conducted on their verbal productions. Cognitive tests assessing verbal episodic memory, executive functions, naming and oral syntactic comprehension were also performed. Brain gray matter correlates of the ND comprehension test were examined using voxel-based morphometry (VBM). An ND comprehension impairment was found for prodromal and mild DLB patients as compared to controls. These difficulties were correlated with the Frontal Assessment Battery (FAB) score. ND comprehension impairment in DLB was further characterized by a deficit in the organization and the logic of the discourse. Moreover, VBM analysis revealed a correlation between striatal gray matter volumes and DLB patients’ ability to extract and organize relevant information (*p* < 0.05, FDR correction, cluster level). The ND comprehension impairment in DLB patients could be related to their executive dysfunction through a deficit of information selection and organization that correlates with the volumetric reduction of striatal gray matter.

## Introduction

Verbal comprehension is a complex interpretative activity that leads to the conception of coherent mental representations. Comprehension implies not only linguistic abilities but also attention, memory, pragmatics and executive skills (Kim et al., 2019). Coherence is guaranteed by theme maintaining, adequacy between information and the congruence between mental representation and personal knowledge. The coherent representation of a discourse is ensured by the generation of inferences (Silagi et al., 2014). Coherence is established on two levels; local coherence corresponds to the microstructure of the discourse, and global coherence corresponds to its macrostructure (Kintsch, 1988). Most importantly, the continuous integration of both local and global coherence in a situational model integrating one’s general knowledge about the world and context is required for a complete comprehension of the discourse.

At the brain level, several regions have been suggested as supporting discourse comprehension beyond the left dorsomedial prefrontal cortex (Ferstl et al., 2007). The processing of narrative coherence would be specifically associated with cingulate cortex, medial frontal (Ferstl et al., 2007) and parietal regions, while posterior parietal regions would be involved in the construction of the situational model, and frontal-temporal networks would support its maintenance over time (Yarkoni et al., 2008). More specifically, inference elaboration would rely on temporal regions (Virtue et al., 2006; Kim et al., 2019).

Discourse comprehension implies numerous cognitive abilities that may be impaired during aging, which is why a growing number of studies have investigated the evolution of comprehension in aging. Indeed, an aging effect is observed on inference generation in healthy adults (Williams et al., 2012). In neurodegenerative diseases, comprehension is further reduced (Murray and Stout, 1999; Welland et al., 2002; Monetta et al., 2009; Gaudreau et al., 2013). Detail and implicit information processing, like inferences, is impaired in early stage Alzheimer’s disease (AD; Welland et al., 2002; Gaudreau et al., 2013), Parkinson’s disease (PD; Murray and Stout, 1999; Monetta et al., 2008, 2009) and Huntington’s disease (HD; Murray and Stout, 1999; Saldert et al., 2010). However, to date, little is known about narrative discourse (ND) comprehension in dementia with Lewy bodies (DLB).

Dementia with Lewy bodies is a cognitive neurodegenerative disease considered as the second leading cause of dementia, representing 15–20% of all dementia. DLB diagnosis is characterized by cognitive impairment plus the presence of two of the core clinical features, namely attentional fluctuations, visual hallucinations, REM sleep behavior disorder and elements of parkinsonism (McKeith et al., 2017). At the cognitive level, DLB impacts several functions: patients display cognitive and attentional fluctuations (Ballard et al., 2001, 2002), an early visuoconstructive impairment (Kemp et al., 2017), an executive dysfunction characterized by a lack of flexibility and thought mobility (Petrova et al., 2015; Kemp et al., 2017) and a moderate alteration of semantic memory as compared to AD (Calderon et al., 2001). At the brain level, Lewy body spectrum disorders (LBSD) such as PD, Parkinson’s disease with dementia (PDD) and DLB are characterized by diffuse brain lesions related to the progression of the abnormal aggregation of alpha-synuclein, associated with pervasive cellular death in dopaminergic regions of the brain. However, contrary to other synucleinopathies, like PD, the pattern of alteration seems to spread to more cortical regions, such as the insula (Roquet et al., 2017), and to be associated with both phosphorylated tau and beta-amyloid deposits in the affected regions (Patterson et al., 2019).

Studies investigating language in DLB are scarce and tend to include other LBSD, such as PD and PDD. It has, however, been demonstrated that DLB patients have a reduced speech rate as compared to PD, PDD and control subjects, especially when the syntactic complexity increases, independently of language skills and possibly due to an executive dysfunction (Ash et al., 2012b). Moreover, it has been demonstrated that although the lexicon seems to be preserved in LBSD (Ash et al., 2012a; Boschi et al., 2017), action fluency would be particularly impaired in DLB (Delbeuck et al., 2013), suggesting an alteration of syntactic processes. Accordingly, in LBSD, and more particularly in DLB, studies found a syntactic impairment in both production (Ash et al., 2012a; Boschi et al., 2017) and comprehension, characterized by a deficit in syntactic ambiguity processing (Grossman et al., 2012). More generally, the organization of ND would be impaired in LBSD: the appreciation of the internal structure of scripts, theme maintaining, and coherence are disturbed. These deficits would be related to the executive dysfunction (Ash et al., 2012b; Gross et al., 2013; Grossman et al., 2017). Indeed, Ash et al. (2012a) found frontal and temporal cortical atrophy to be linked to the judgment of script organization. This led them to propose the involvement of the frontal ventrolateral cortex in ND coherence given that this region is involved in both executive functioning and ND. Moreover, Gross et al. (2013) highlighted the role of basal ganglia and frontal-striatal networks in temporal sequencing and selection of competing actions processes, which led them to suggest the potential contribution of a subcortical dysfunction in the script processing impairment in LBSD.

Accordingly, the goal of this study was to investigate the link between early executive dysfunction in DLB and discourse comprehension impairment. We predicted that early stage DLB patients would be more impaired in a narrative comprehension task than control subjects. The DLB narrative deficit would be related to DLB patients’ executive dysfunction through the disorganization of ND. Moreover, the ND comprehension deficit would be supported by a gray matter loss in frontal regions, which support executive functioning.

## Participants and methods

### Participants

Forty-five older adults participated in the study, comprising 26 patients with probable DLB and 19 controls. The experimentations were conducted in Strasbourg at geriatric day hospital of the Hôpital Saint François (France) from September 2018 to March 2019. All participants were fluent French speakers, free from any non-compensated hearing loss or language impairment that would impede comprehension of instructions, and free from noticeable anomia (see below for details of neuropsychological testing). DLB participants were included as follows: diagnosis of probable DLB according to the criteria of McKeith et al. (2017) for mild DLB and McKeith et al. (2020) for prodromal DLB, Mini-Mental State Examination (MMSE) score superior or equal to 19 in the absence of a comorbid neurodegenerative disorder. Controls were excluded if they presented an MMSE score below 26, a subjective cognitive complaint or a history of brain disease.

### Procedure

All participants underwent a neuropsychological examination on the day of testing. Global cognitive functioning was assessed by MMSE, oral syntactic comprehension by GREMOTs (Bézy et al., 2016), image denominations by DO80 (Deloche and Hannequin, 1997), episodic memory by the French version of the free and cued selective reminding test (FC-SRT) called RL-RI-16 (Van der Linden et al., 2004), and executive functioning by Frontal Assessment Battery (FAB; Dubois et al., 2000).

Narrative discourse comprehension was assessed using the ND test taken from the Montreal Protocol for the Evaluation of Communication (MEC; Joanette et al., 2004), which is aimed at investigating inferential processes involved in access to the meaning of the story. In this test, a 214-word-long story consisting of 5 paragraphs (corresponding to the narrative structure; initial situation, complications, transforming action, denouement, final situation) is read to the participant twice. The first reading is fragmented by paragraph and participants are asked after each paragraph to immediately recall as many elements as they can. Thirty elements are expected to be recalled for the whole story (total information score/30), out of which 17 are considered to be principal elements (principal ideas score/17). The second reading is performed in its entirety without pause, after which the participant is instructed to recount the story, out of which 13 key elements are expected (entire recall score/13). Finally, participants are asked 12 comprehension questions to estimate their global understanding of the story (comprehension questions score/12).

A qualitative grid, inspired by several narrative analysis grids (Boutard Corinne, 2010; Bézy et al., 2016), was created to evaluate recall coherence. It assesses the reconstruction of the narrative by the participant throughout the story recall. Each point evaluated in this grid is representative of narrative coherence and involves executive functioning. The grid is comprised of 7 sub-items, distributed in 4 groups:

– Narrative theme.
– Discourse organization: respect of narrative structure, actions chronology and main ideas/details proportion.
– Logic of discourse: sequence of actions and relation markers.
– Discourse coherence: references.

Each sub-item was evaluated using ordinal scale from 0 (highly impaired) to 3 (normal performance). A narrative coherence score (out of 21) was finally obtained. Each examination was recorded and transcribed in order to evaluate the productions.

### Neuroimaging

Whole-brain isotropic high-resolution T1-weighted MPRAGE images (TR = 1.9 s; Flip angle = 9°; TE = 2.53 ms; TI = 900 ms; FOV = 192 × 192 × 176; voxel size = 1 mm^3^) were acquired for 23 of the 26 DLB patients during the same day as the behavioral testing using a 3T Siemens Magnetom VERIO MRI (Siemens, Erlangten, Germany) equipped with a 32-channel coil.

Data analysis was carried out using the Statistical Parametric Mapping package (SPM12; Wellcome Trust Centre for Neuroimaging). Briefly, after visual inspection and quality control, the images were segmented into 6 different maps of tissue class probabilities, including gray and white matter. These maps were subsequently spatially normalized using non-linear deformations (DARTEL algorithm) to obtain a study-specific template, which was in turn coregistered to the MNI152 template. The DARTEL flow fields were subsequently used to create a normalized gray matter (GM) map for each participant, in which the voxel-wise value of GM probability was modulated depending on the amount of warping applied during normalization in order to preserve global GM quantities, and smoothed using an 8 mm^3^ full width at half-maximum (FWHM) Gaussian filter. Statistical analysis was then carried out on DLB patients’ brain MRIs using a general linear model GLM model including 4 regressors of interest (principal ideas, total information, entire recall and comprehension questions) and a regressor of non-interest (total GM volume), with an uncorrected voxel-wise threshold of *p* < 0.005, cluster-wise corrected at *p* < 0.05 false discovery rate FDR.

### Statistical analysis

The group differences between the control participants and the DLB participants, concerning ND and narrative coherence, were evaluated using Student and Wilcoxon-Mann-Whitney statistical tests (significance level set at 0.05). Statistical correlations were evaluated in the DLB group between narrative discourse score, narrative coherence score, MMSE, age and neuropsychological tests using Pearson’s and Spearman’s correlation tests (for interval and ordinal scale variables, respectively).

## Results

### Participants’ characteristics

The two groups, DLB and controls, were not statistically different regarding age, sex, oral syntactic comprehension score and memory scores (Table 1). However, as expected, there was a significant difference between the two groups for the MMSE, DO-80 and FAB scores, with DLB participants performing less well than controls.

**TABLE 1:** Comparison of participants’ characteristics between the DLB and control groups.

Characteristics	DLB group	Control group	Statistics	*P*-value
Group (*n*)	26	19		
Age *(sd)*	71.15 *(10.43)*	66.37 *(8.58)*	T (43) = −1.63	0.109
Sex ratio (F/M)	15–11	13–6	Chi^2^ (1) = 0.54	0.463
MMSE *(sd)*	26.08 *(3.02)*	28.80 *(1.13)*	T (43) = −3.72	0.00057*
Oral syntactic comprehension *(sd)*	5.85 *(0.61)*	6 *(0)*	T (43) = −1.09	0.281
DO80 *(sd)*	76.64 *(4.25)*	79.42 *(0.84)*	T (42) = −2.80	0.0076*
FAB *(sd)*	15 *(2.91)*	17.47 *(0.96)*	T (43) = −3.55	0.0009*
Free total recall–RL-RI/16 *(sd)*	24.23 *(8.79)*	28.05 *(6.60)*	T (39) = −1.56	0.128
Delayed total recall–RL-RI/16 *(sd)*	14.65 *(2.48)*	15.5 *(1.20)*	T (36) = −1.32	0.196

F/M, female/male; MMSE, mini–mental state examination; FAB, frontal assessment battery.

*Indicates significance at *p* < 0.05.

### Intergroup comparisons

Globally, DLB patients performed worse than control participants in the ND tasks. DLB participants recalled less information than controls [principal ideas score T (43) = 3.37; *p* = 0.002 and total information score T (43) = 3.17; *p* = 0.003].

The entire recall and comprehension mean Z-scores of DLB patients were lower than those of controls [entire recall score T (43) = 2.91; *p* = 0.006 and comprehension questions T (43) = 3.76; *p* = 0.0005], with a marked variability of the DLB performances; Thirty-one percent of DLB patients were within the pathological range concerning entire recall, and 38.5% of them exceeded the pathological cut-off concerning comprehension questions.

Narrative coherence scores were significantly lower in the DLB group than in the control group (*U* = 83; *p* = 0.0003).

**FIGURE 1:**
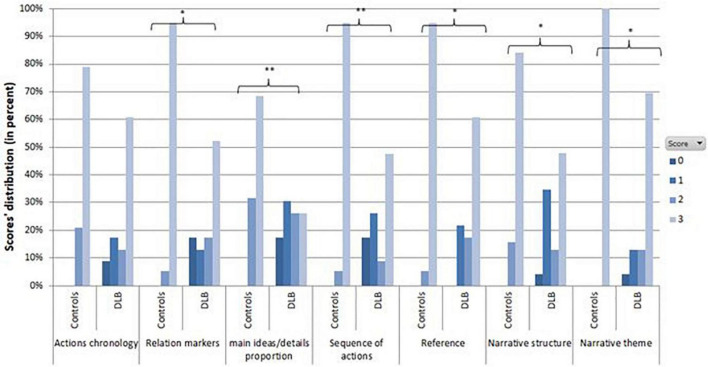
Distribution of each sub-item of narrative coherence in DLB patients and controls (Significance: *p < 0.05; **p < 0.001).

In terms of “actions chronology,” there was no statistical difference between the DLB and control groups (*U* = 167; *p* = 0.114). DLB patients obtained significantly lower scores than controls on “narrative themes” (*U* = 152; *p* = 0.0101), “respect of narrative structure” (*U* = 125.5; *p* = 0.006), “relations markers” (*U* = 122; *p* = 0.002) and “references” (*U* = 142; *p* = 0.0099). DLB group scores were much lower than those of the control group for “main ideas/details proportion” (*U* = 93; *p* = 0.0008) and “sequence of actions” (*U* = 111; *p* = 0.0009) (Figure 1).

In the control group, narrative coherence scores were homogeneous and tended to reach the maximum, with most points lost in the “main ideas/details proportion” section (Figure 1). In the DLB group, narrative coherence impairments were particularly present in 3 sub-items: “main ideas/details proportion,” “respect of narrative structure,” and “sequence of actions” (Figure 1).

### Clinical correlations

In the control group, no statistical correlation was observed between the scores of the ND test and the neuropsychological examination. In the DLB group, the entire recall score correlated significantly with the MMSE score [r(24) = 0.61; *p* = 0.00098], age [r(24) = −0.5; *p* = 0.01] and with the free total recall of RL-RI/16 [r(20) = 0.61; *p* = 0.0026]. The comprehension questions score correlated with the MMSE score [r(24) = 0.5; *p* = 0.009] and with the entire recall of the ND test of the MEC protocol [r(24) = 0.696; *p* = 0.000079]. There was no significant correlation between the comprehension questions score and age [r(24) = −0.38; *p* = 0.055], the FAB score [r(24) = 0.32; *p* = 0.11] or the total delayed recall of RL-RI/16 [r(18) = 0.09; *p* = 0.70]. The qualitative narrative coherence score correlated with the FAB score [r(21) = 0.5; *p* = 0.014]. The Principal ideas score of the MEC protocol correlated with the FAB score [r(24) = 0.52; *p* = 0.007] but did not significantly correlate with the total free recall of RL-RI/16 [r(20) = 0.29; *p* = 0.19].

Those DLB patients who did not produce the final inference performed significantly lower at comprehension questions than the DLB patients who produced the final inference (*U* = 12; *p* = 0.0005), suggesting a link between a high score on comprehension questions and the generation of the final inference. There was no statistical difference between DLB patients producing the final inference and those not producing it in terms of the FAB score (*U* = 60; *p* = 0.382).

### Voxel-based morphometry correlations

**FIGURE 2:**
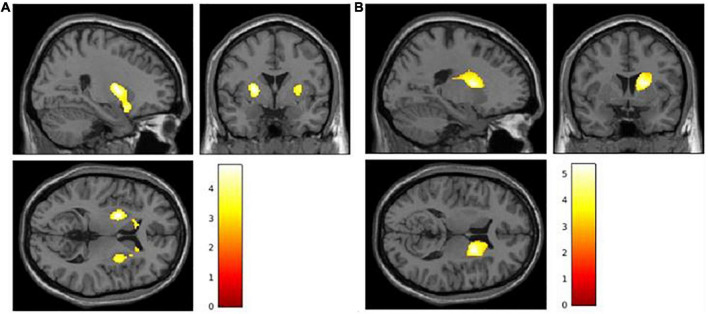
**(A)** Correlation between gray matter volume and principal ideas score. **(B)** Correlation between gray matter volume and entire recall Z-score. Colored voxels show regions for which statistical analysis is significant at *p* < 0.005 at the voxel level and FDR-corrected at *p* < 0.05 at the cluster level.

Gray matter density in bilateral putamen and bilateral caudate nuclei (i.e., bilateral striatum) was found to be positively correlated in DLB participants with their principal ideas score (*p* < 0.05, cluster-wise FDR-corrected, extend threshold 1889 voxels; Figure 2A). Additionally, a positive relationship was found between the gray matter volume in the right caudate nucleus and the entire recall Z-score in DLB participants (*p* < 0.05, cluster-wise FDR-corrected; Figure 2B). The correlation between the entire recall and the caudate nuclei becomes bilateral when the cluster-wise threshold is lowered (extend threshold 500 voxels), which seems to indicate that the left caudate nucleus is also involved. Other variables (total information score and comprehension Z-score) did not display a significant correlation with the gray matter volume of either of these cerebral areas.

## Discussion

The aim of the present study was to investigate to what extent the executive deficit associated with the early stage of DLB would influence the comprehension of ND in DLB patients. Our results confirmed an impairment of narrative comprehension in DLB patients and a strong correlation between their level of executive functioning and both their narrative coherence and principal ideas scores, which indicates that their capacity to re-elaborate the narrative meaning could be related to the efficiency of their executive functioning. The impairment of narrative comprehension further appeared related to a lower volume of gray matter in the striatum of patients.

Several studies have also shown deficits in narrative organization between DLB patients. Gross et al. (2013) found that DLB and PDD patients were less sensitive to the events’ order of a script than PD patients and controls, which highlights the deficits of DLB and PDD patients in processing the structure of a script. Likewise, Ash et al. (2012b) demonstrated a reduced sensitivity to the hierarchical organization of a narrative in DLB and PDD patients, as compared to both controls and PD patients. Interestingly, in their study, theme maintaining and connections between items, which rely the most on executive functioning, were also the most affected domains. In addition, the narrative organization was more impaired in DLB subjects than in PDD subjects. Ash et al. (2012a) concluded that these difficulties in the comprehension and production of the organization of the narrative result from an executive control deficit.

Accordingly, our qualitative findings indicate that patients in the DLB group were particularly impaired on the sub-items “main ideas/details proportion” and “sequence of actions,” indicating that their profile is characterized by a deficit in prioritizing and organizing information. Thus, DLB patients’ dysexecutive syndrome could be involved in their deficit of selection and organization of relevant information affecting the link between microstructure and macrostructure, and ultimately coherence. Accordingly, DLB patients show difficulties with both local and global coherence processing as compared to control subjects (Boschi et al., 2017).

We expected that the narrative comprehension deficit would be related to frontal gray matter loss. Indeed, the narrative comprehension literature highlights the implication of the dorsomedial prefrontal cortex as an essential neural substrate of coherence processes (Ferstl et al., 2007; Yarkoni et al., 2008; Ash et al., 2012b; Noh et al., 2014). Difficulties in narrative discourse in neurodegenerative diseases such as corticobasal syndrome are reported to be related to atrophy patterns in frontal and parietal regions compatible with executive functioning (Gross et al., 2010). In the latter study, the deficit in narrative discourse was underlain by an alteration of the integration of the information into a meaningful whole, which is central to the entire recall and relies heavily on multiple round trips between the micro and macro level. These elements are congruent with our own behavioral results since the principal idea and entire recall scores involve the selection of relevant information through the same processes.

Our volumetric analyses did not find any significant link with cortical regions’ gray matter density, but rather revealed a correlation between the gray matter density in the striatum of DLB participants and their principal ideas score as well as their entire recall score, more specifically in the bilateral putamen and the right caudate nucleus, respectively. Although the role of the striatum in motor control is now well accepted, several studies have highlighted the implication of the putamen and the caudate nucleus in language processes such as speech (Viñas-Guasch and Wu, 2017), syntax (Teichmann et al., 2006; Long et al., 2019) and high-order language processes (Moretti et al., 2017; Viñas-Guasch and Wu, 2017). Most importantly, Moretti et al. (2017) reported that basal ganglia enhance cortical efficiency in language processing, especially through their role in selecting relevant signals. Indeed, functional models of basal ganglia describe the extensive functional interconnection between the striatum and the prefrontal cortex (PFC; Monchi et al., 2001, 2006; Moretti et al., 2017).

Simard et al. (2011) distinguished two different loops participating to the executive processing of verbal material: one involved in planning processes, implicating the caudate nucleus and the ventrolateral PFC, and the other, involved in execution processes, implicating the putamen and the posterior PFC. Furthermore, according to Provost et al. (2010), although all monitoring processes (both self-ordered and externally triggered) involve the dorsolateral PFC, only self-ordered monitoring specifically requires the caudate nucleus. Finally, Gross et al. (2013) assumed the implication of striato-frontal networks and the basal ganglia in temporal sequencing and competing actions selection. Overall, our results therefore suggest a link between the alteration of striatal regions involved in specific executive components of language processing as a source for narrative comprehension impairment in early DLB patients.

Accordingly, we found correlations between some qualitative indicators of narrative comprehension and behavioral measures of executive functioning. However, we assessed the latter as a whole using a screening test (FAB), which did not allow for an exploration of specific executive components involved in the effect. Future studies should therefore include various executive tests such as the Trail Making Test (TMT) or Stroop test in order to investigate this matter more thoroughly.

Moreover, our samples were relatively small, and there was no other degenerative disease group that would have allowed us to assert the specificity of this link. We thus cannot rule out the possibility that the overall cognitive impairment resulting from dementia is not responsible for the alteration of ND. Grossman et al. (2017) argued that LBD patients have more difficulties in assessing ND than non-demented LBD patients, indicating a progression of ND alteration throughout the development of the disease. Nonetheless, these difficulties in narrative organization were not correlated with the level of dementia (MMSE score), neither could they be entirely explained by a linguistic deficit.

Additionally, given that the deficit in ND was found on all of the subscores of the MEC scale, one could argue that the poor performance in ND was related to memory deficits. However, our participants did not display an impairment of episodic memory according to their scores in RL/RI 16, which tends to indicate that these narrative comprehension difficulties were not particularly due to verbal episodic memory deficits.

Another limit of our study is that we did not exclude patients with a psychiatric onset of prodromal DLB, even though all of our prodromal DLB patients had MCI. It is of relevance, as the affection of nigrostriatal pathways in either conditions (cognitive or psychiatric onset) can be different (Hansen et al., 2021), possibly in relation with a malfunction of the locus coeruleus (Hansen, 2021) in psychiatric onset patients. Since the locus coeruleus modulates executive functions through the noradrenergic system, and psychiatric symptoms are frequent at the prodromal stage of DLB (Blanc et al., 2022), future studies should explore potential implications of the type of onset of DLB (that is, cognitive, psychiatric or mixed) in this matter. Overall, our results are therefore consistent with the hypothesis that executive dysfunctioning in DLB patients could lead to difficulties in planning and reasoning about the narrative structure. We have demonstrated an impairment of narrative understanding in prodromal and mild DLB patients. Moreover, and contrary to our expectations, we have shown a strong correlation between these difficulties in narrative comprehension and the gray matter density of the striatum. This would appear to be the first demonstration of such a correlation. According to Yang et al.’s (2019), comprising a meta-analysis of 78 functional magnetic resonance imaging (fMRI) studies, discourse comprehension involves the activation of large cortical networks. These different results could be explained by the experimental population characteristics. In our study, striatum involvement in discourse comprehension would likely be linked to DLB pathology. Future research involving functional imaging in prodromal DLB will be needed to go further in the understanding of the role of the striatum and striato-frontal processes underlying narrative comprehension.

## Data availability statement

The raw data supporting the conclusions of this article will be made available by the authors, without undue reservation.

## Ethics statement

This research involving human participants were approved by the ethics committee: Comité de protection des Personnes Est IV Strasbourg (number: Eudract 2012-A00992-41/HUS5330). All participants provided written informed consent to this study.

## Author contributions

AF, MJ, FB, and EF contributed to the conception and design of the study. AF, MJ, LL, PL, and MM organized the database. AF, MJ, and LL performed the statistical analysis. AF and MJ wrote the first draft of the manuscript. LL and FB wrote sections of the manuscript. AF, MJ, LL, and FB contributed to manuscript revision, read, and approved the submitted version. All authors contributed to the article and approved the submitted version.

## Abbreviations

ND: narrative discourse
GM: gray matter
MEC: Montreal protocol for the evaluation of communication
FAB: frontal assessment battery.
